# Editorial: Food-derived polyphenols: functional regulation in chronic diseases

**DOI:** 10.3389/fnut.2024.1333459

**Published:** 2024-01-24

**Authors:** Zhe Xu, Zhijian Tan, Jianbo Xiao, Shuzhen Cheng, Hui Chen

**Affiliations:** ^1^College of Life Sciences, Key Laboratory of Biotechnology and Bioresources Utilization, Dalian Minzu University, Ministry of Education, Dalian, China; ^2^Institute of Bast Fiber Crops and Center of Southern Economic Crops, Chinese Academy of Agricultural Sciences, Changsha, China; ^3^Department of Analytical Chemistry and Food Science, Faculty of Food Science and Technology, University of Vigo, Orense, Spain; ^4^School of Food Science and Technology, National Engineering Research Center of Seafood, Collaborative Innovation Center of Seafood Deep Processing, Dalian Polytechnic University, Dalian, China; ^5^College of Food Science and Technology, Key Laboratory of Marine Fishery Resources Exploitation and Utilization of Zhejiang Province, Zhejiang University of Technology, Hangzhou, China

**Keywords:** polyphenols, chronic diseases, nutrition, absorption, functional regulation

Chronic diseases-such as cancer, diabetes, neurodegenerative diseases, bone diseases, parkinsonism, and hypertension, are the leading reasons for disability and death in the world (1). Many chronic diseases are linked to lifestyle choices and dietary habits. It has been observed that balanced nutrition and healthy dietary patterns can reduce morbidity and mortality of chronic diseases (2). The beneficial effects for health of food-derived bioactive compounds are gradually recognized. Among them, polyphenols show potential antioxidative, anticancer, anti-hypertensive, osteogenic, and anti-inflammatory effects (3), which are considered functional nutraceuticals in preventing chronic diseases (Figure 1). On the other hand, researches have also revealed that the functional qualities of food-derived components are related to processing, preservation, and digestion procedures (4). In addition, food-derived polyphenols can be metabolized via different pathways and eventually absorbed in the body to exert their efficacy (5). However, the research to identify and collect functional components in foods and to investigate their potential effects are still at the beginning (6). Therefore, this Research Topic aims to gather the latest researches about food-derived polyphenols, which can improve physical function and even prevent chronic diseases. The development of differentiated capture strategies for food-derived components and the elaboration of their changes in the preservation or diet will lay the foundation for the in-depth utilization of functional foods.

**Figure 1:**
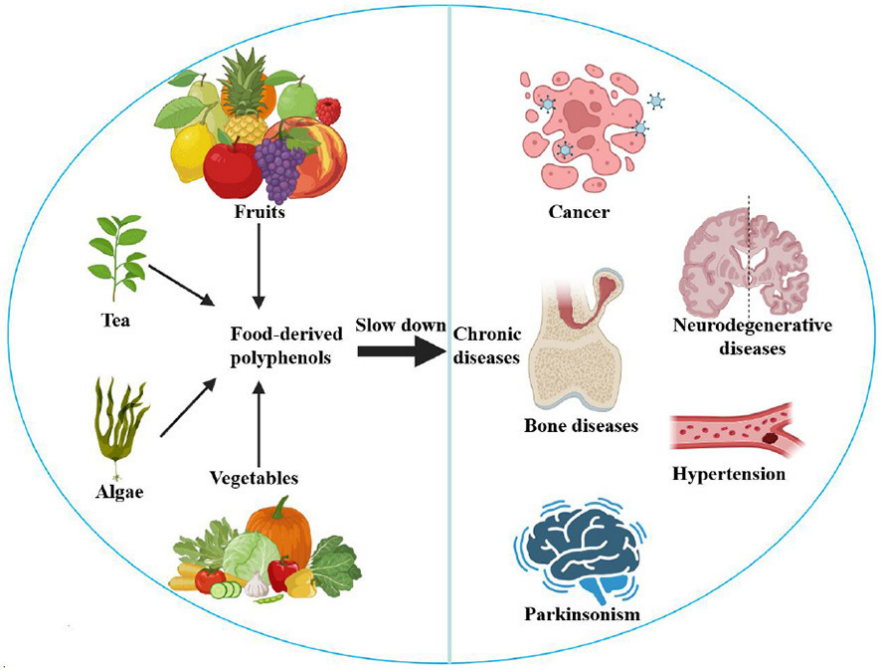
Effects of food-derived polyphenols on chronic diseases.

A total of fourteen submissions have been received for this Research Topic, six of them have been published. The number of manuscripts received indicates that the topic of chronic disease is a key research area. Therefore, the six published articles are briefly summarized.

In these articles for this Research Topic, many compounds were identified that could reduce chronic diseases. Among them, Daidzein, a soy polyphenol, which could reduce metabolic associated fatty liver disease, control attenuation parameter, hepatic steatosis index, and fatty liver index to improve hepatic steatosis from 1,476 participants in National

Health and Nutrition Examination Survey (NHANES) and the Dietary Studies of USDA Food and Nutrient Database (Yang et al.). Polyphenols from *Acanthopanax acanthopanax* extract were determined to mediate phagocytosis in macrophages and monocytes through the Fcγ receptor signaling pathway. They also influenced the PI3K/AKT signaling pathway and the insulin receptor signaling pathway, which slowed Parkinson's disease in mice (Li et al.). Additionally, Bai et al. demonstrated that phlorotannin from *Ascophyllum nodosum* could stably combine with phycocyanin by ultrasound treatment and had the potential to reduce light damage in Retinal Müller cells. *Ascophyllum nodosum* and *Camellia sinensis-leaf* extract was able to reduce levels of glucose, adiponectin, leptin, and the inflammatory factors IL-1β and TNF-α, and jointly interfere with the glucose lipid and energy metabolism in obese mice (Xu et al.). Upadhyay et al. demonstrated that PL02 containing phenolic substances was a potential drug for relieving osteoarthritis by down-regulating CGRP1, COX-II and MMP13, and up-regulating BCL2, SOX-9 and COL-1 proteins in rats. In addition, some active substances in diabetic patients had some applications such as pure buckwheat noodles adding with calcium and sodium alginate, which indicated the possible presence of an amylose-lipid complex (V-type) in the starch particles and endowed the starch with properties such as resistance to digestion *in vitro* and improved food texture (Wang et al.). However, most of the above reports still remain at study of mixtures, and some explanations of the prevention and control mechanism on chronic diseases not yet fully elaborated. Although these food-derived substances have shown promising progress in the prevention and treatment of chronic diseases, further experiments are needed to explore confounding factors and in-depth mechanisms. The bioavailability of these food-derived active substances was also not reflected in these studies. Therefore, it is necessary to further screen out the pathway and bioavailability of key single active ingredients for animal experiments and clinical trials in order to strengthen the prevention and treatment mechanism of chronic diseases.

In conclusion, this Research Topic can help researchers better understand the influence of food-derived polyphenols on chronic diseases. In addition, this Research Topic discussed the mechanism of some food-derived substances on chronic diseases, and proposed some effective components. However, the bioavailability of the food-derived substances *in vivo* needs to be further determined, and the molecular mechanisms of these components prevent chronic diseases need to be further explained.

## Author contributions

ZX: Formal analysis, Funding acquisition, Writing – original draft. ZT: Writing – review & editing. JX: Writing – review & editing. SC: Writing – review & editing. HC: Writing – review & editing.
